# Descriptive Epidemiology of Brain and Central Nervous System Tumours: Results from Iran National Cancer Registry, 2010-2014

**DOI:** 10.1155/2020/3534641

**Published:** 2020-09-18

**Authors:** Amir Salimi, Alireza Zali, Amir Saeid Seddighi, Afsoun Seddighi, Shakila Meshkat, Morteza Hosseini, Amir Nikouei, Mohammad Esmaeil Akbari

**Affiliations:** ^1^Cancer Research Center, Shahid Beheshti University of Medical Sciences, Tehran, Iran; ^2^Functional Neurosurgical Research Center, Shahid Beheshti University of Medical Sciences, Tehran, Iran; ^3^School of Medicine, Tehran University of Medical Sciences, Tehran, Iran

## Abstract

**Background:**

Central nervous system (CNS) tumours account for only 1-2% of cancer incidence but are a major reason for mortality and morbidity due to malignancies. Recent studies show an increase in the rate of CNS tumours worldwide, especially in developing countries. Moreover, there is significant heterogeneity in epidemiological patterns worldwide. This study is aimed at representing nationwide epidemiology of CNS tumours in Iran.

**Methods:**

Iran National Cancer Registry 2010-2014 data were reviewed for CNS tumours. The epidemiological rates were calculated for both genders and all age groups using the 2011 census information.

**Results:**

Out of 17345 cases, 58.5% were men and 41.5% were women. The mean age was 45.55 years ranging from less than 1 month to 100 years old. Average total age-standardized incidence rate (ASR) was 5.19 for primary tumours. The annual percent change (APC) was 14.23% during the study period. The most frequent site and histology recorded were brain, NOS and diffuse astrocytic, respectively. Geographical distribution showed about five-fold difference in ASRs between different provinces.

**Conclusion:**

The overall ASR calculated was higher than the global rate in 2012 but lower than that of most developed countries, showing an increasing trend which may be due to either advances in diagnosing or risk factor augmentation. The mean age and incident rates were higher than those of previous reports in Iran.

## 1. Introduction

Central nervous system (CNS) tumours are much less prevalent, accounting for only 1-2% of the total cancer incidence. However, they represent a major source of mortality and morbidity [1–3]. In the United States, CNS cancers represent 1.4% of newly diagnosed cancers and 2.7% of cancer mortality [4]. Recent studies show an increase in the prevalence of CNS tumours worldwide [2, 5]. Based on the most recent estimates, each year, more than 296000 new cases (1.6% of overall cancer incidence) are diagnosed worldwide, and more than 241000 individuals die due to CNS tumours (2.5% of overall cancer mortality) [1, 6, 7] with men showing a higher incidence rate (IR) (4.2 in 100000 inhabitants) than that of women (3.6 in 100000 inhabitants) [6]. The IR of all primary CNS tumours ranges from 17.6/10^5^ to 22.0/10^5^ in America [8] and Europe [7, 9, 10].

Central nervous system tumour is typically defined as brain and spine tumours; however, the International Classification of Diseases for Oncology third edition (ICD-O-3) defines meninges, pineal gland, pituitary gland, and nerves as CNS tumours as well [11].

There is significant heterogeneity in the epidemiology, incidence, mortality, and histological spectrum of CNS tumours across different regions of the world [3, 12]. The probable reason for such differences in epidemiologic patterns is yet unclear, but differences in environment, genetics, culture, age, and access to health resources could be considered [1, 2]. Comprehensive and systematic epidemiological studies can further our knowledge of such influences worldwide.

Developed countries conducted a number of comprehensive epidemiological studies on CNS tumours [5, 8, 13–17]. In contrast, in developing countries including Iran [18–20], scattered studies have been conducted. There are not any comprehensive nationwide studies in this area [21–28]. Thus, in the current study, we aimed to present a nationwide CNS tumour epidemiology for all age groups in all provinces of Iran as well as the epidemiologic trend during the study period based on Iran National Cancer Registry (INCR) data.

## 2. Material and Methods

### 2.1. Data Source

The study included patients who were diagnosed with central nervous system cancer from 2010 to 2014. The patients were identified by ICD-O-3 codes (Tables 1 and 2) through the Iran National Cancer Registry. National cancer registry raw data were reviewed for all registered CNS tumours from 2010 to 2014. Based on histology reports, some of the patients were categorized as “secondary tumours” and “undetermined secondary or primary” but most of the patients were categorized as “primary tumours” and we focused on them in our study. Reporting benign CNS tumours is not compulsory and systematic yet, but if reported by a center, they would be involved in the INCR data.

### 2.2. Design

We conducted a nationwide population-based study on national cancer registry data on CNS tumours over 5 years from 2010 to 2014. Duplicate cases were first identified according to patients' names, family, tunour type, tumour location, and sex, then were excluded from the study. Data were categorized based on WHO classification of CNS tumours histologies reported by Louis et al. [29]. Population data were obtained from the last 2011 census On the National Institute of Statistics of Iran webpage (https://amar.org.ir/), and the Segi-Doll standard population data were obtained from The Global Cancer Observatory (GCO) website [30] to perform ASR calculations.

### 2.3. Statistical Analysis

Categorical data are presented as numbers and percentages. To compare categorical and continuous data between male and female groups, chi-squared and independent-sample *t*-tests were applied.

The last 2011 census of population information was used to calculate (age- and gender-specific) crude incidence rates (CIRs) per 100000 individuals. To obtain annual incidence rates from 2010 to 2014, the estimated midyear population was used as denominator. The Segi-Doll standard population data were used to calculate age-standardized rates (ASRs) by the direct standardization method.

The incidence rates are presented with 95% confidence interval (95% CI). To investigate the trend of the rates during this period of time, a log-linear model was fitted and the average annual percent change (APC) was calculated based on the model parameter [31]. All calculations and statistical analysis were carried out using Microsoft Excel 2013 and R statistical software, version 3.2.1. The significance level was set at 0.05.

### 2.4. Ethical Approval

This study was approved by the Shahid Beheshti University of Medical Sciences Ethics Committee.

## 3. Results

### 3.1. Overall Incidence

In this study, we reviewed 17345 incident cases of CNS tumours from 2010 to 2014. Only 50.4% of data were microscopically verified (MV). Out of 17345 cases, 10151 were men (58.5%) and 7194 were women (41.5%).

Based on the nature of tumours, we divided them into three major groups: (i) primary, (ii) secondary, and (iii) undetermined whether secondary or primary (SorP). The mean age at diagnosis for patients in the primary, secondary, and undetermined groups was 45.55 ± 21.46, 49.78 ± 19.17, and 35.44 ± 21.95, respectively, with a statistically significant difference between them (*p* value < 0.001).

The highest average ASR during the study period was observed among primary tumours (5.19; 95% CI (4.99-5.38)) in both men (5.56 (5.41-5.71)) and women (3.93 (3.81-4.06)). Secondary and secondary or primary (SorP) had ASRs of 0.21 and 0.04, respectively. Annex 1 shows the number of patients in the study period in each age category separated by gender. Patients under 1 year of age are presented separately due to the importance of CNS tumours in this group of patients. Annex 2 depicts the annual age-specific CIR and the number of patients per year from 2010 to 2014 and the average CIR during the entire study period. Hereafter, all rates and reports are for 16547 primary tumour cases.

Mean age at diagnosis was 45.54 (SD = 21.45); the oldest patient was 100 years old and the youngest patient was a neonate. Out of 16547 cases, only 8115 (51.1%) were microscopically verified, 9695 (58.6%) were male, and 6852 (41.4%) were female.

### 3.2. Age-Standardized and Gender-Specific Incidence Rate of Primary Tumours

**Figure 1:**
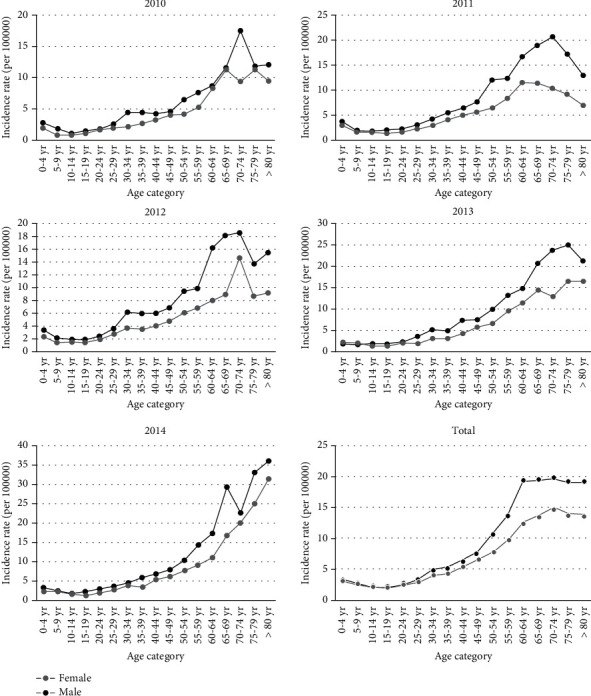
Crude incidence rates by gender and age in each year and the whole period.

Table 3 depicts the annual ASRs and the number of patients per year from 2010 to 2014 and the average ASR during the entire study period. The average ASR was 5.19 (4.99-5.38) per 100000 individuals per year. The highest CIR among age groups was 16.25 (for the 75-79 y group) and the lowest was 1.34 (for the 10–14-year-old age group). The annual CIR and the average CIRs are presented by age group in Annex 2. The annual CIR and the average CIRs were depicted by age group and gender in Figure 1.

The total ASR of primary tumours in the entire study period was 5.19 (4.99-5.38), and the total ASRs were 5.56 (5.41-5.71) and 3.93 (3.81-4.06) for men and women, respectively (Table 3). The ASR increased from 3.43 (3.33-3.54) in 2010 to 5.60 (5.47-5.72) in 2014. According to the Poisson regression model result, a significant rising trend was observed in the total ASR rates during this period (*p* = 0.001 for trend). The annual percent change (APC) was 14.23% during the study period. The ASR changed from 3.96 (3.84-4.07) in 2010 to 6.48 (6.34-6.62) in 2014 for men and from 2.92 (2.82-3.01) in 2010 to 4.76 (4.64-4.88) in 2014 for women. During the study period, there was no statistically significant trend of ASR among men (*p* = 0.211), but an increasing trend of ASR was observed among women (*p* < 001). APC was 14.32% and 14.25% for men and women, respectively.

### 3.3. Tumour Site (Topography)

The most common recorded sites are as follows: brain, NOS; frontal lobe; nervous system, NOS; and temporal lobe. Each comprise 54.2%, 8%, 6.9%%, and 6.3% of all data, respectively. A statistically significant difference was observed between the genders' distribution of tumour location regarding the frontal lobe, temporal lobe, parietal lobe, meninges, and brainstem. Men had significantly more tumours in the frontal lobe, temporal lobe, and parietal lobe, and women had more tumours in the brainstem and meninges (*p* < 0.05). Men with tumours in the occipital lobe were significantly older than women (*p* < 0.05), while women with tumours in the cerebellum were significantly older than men (*p* < 0.05).  Table 4 shows the mean age and number of patients based on tumour location in both genders with a significant difference between females and males in the occipital lobe and cerebellum.

### 3.4. Tumour Histology

The number of patients and ASRs are presented in Table 5. The most common histology subgroups are diffuse astrocytic (48.36%), unspecified malignancy (32.32%), and embryonal tumours (4.55%) with ASRs of 2.40, 2.04, and 0.23, respectively.

### 3.5. Geographic Distribution

**Figure 2:**
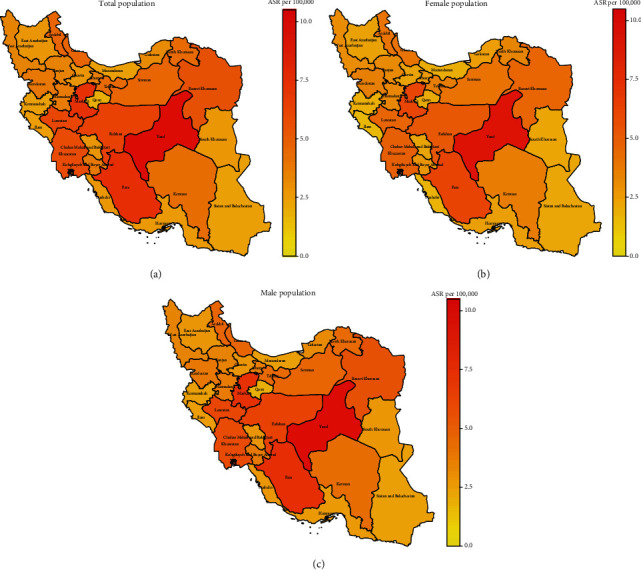
Geographical distribution of CNS tumours by provinces in the total population (a), in the female population (b), and in the male (c) population.

Table 6 and Figure 2 show the geographical distribution of CNS tumours in all 31 provinces of Iran based on their average ASRs. Unlike other provinces, the Qom province showed a female predominance. Kohgiluyeh and Boyer-Ahmad and Bushehr provinces had male to female ratios higher than 2, and Qom showed a female predilection in the number of patients.

## 4. Discussion

In this study, we aimed to provide nationwide descriptive epidemiology of CNS tumours among the Iranian population using INCR data. ASRs were calculated using the Segi-Doll standard population to make comparisons more feasible. Hasanpour-Heidari et al. reported completeness of 96.3% for CNS tumours in Golestan province, which was the best rate among other groups of tumours. Mohammadi et al. reported INCR completeness of 71.2% and 59.9% for men and women, respectively, regarding all types of registered tumours [32, 33].

The average ASR for all primary CNS tumours registered from 2010 to 2014 was 5.19 (4.99-5.38) per 100000 individuals per year, which was higher than the 2012 global rate of 3.4 [31]. However, our national 2014 ASR increased to 5.19, which was higher than the global rate in 2016 but lower than most developed countries' rates [1, 2, 5, 8, 13, 14, 34]. During 2010-2014, average ASRs were 5.56 and 3.93 for men and women, respectively. Miranda-Filho et al. applied data from 96 registries in 39 countries to evaluate CNS tumours in adults (>15 y); Brazil, Croatia, Thailand, and Uganda showed the highest ASR for men (=13.3), highest ASR for women (=12), lowest ASR for men (=2.4), and lowest ASR for women (=1), respectively [35].

CNS tumours' ASR demonstrated an annual percentage change of 14.23% during the study period. This incidence surge and a high APC in Iran like other developing countries could be attributed to advances in neuroimaging, neuropathology, and neurosurgery [36–38] or changes in lifestyle, population pyramid, and stronger registration over time [35, 39]. Other reports from Iran reported high APC rates; Darabi et al. reported an APC of 16.7% and 20% for stomach tumours and colorectal tumours, respectively [40]. Leukemia incidence APC was 19.9% in Koohi et al.'s evaluation [41], and Pakzad et al. calculated an APC of 17.3% for prostate tumours [42].

Higher CT scan utilization leads to more diagnosis, and it also has more ionizing emissions than plain radiographs [43] and shows association with CNS tumours [44]. A recent report showed that the number of CT scans performed per each emergency room visit in an Iranian hospital in 2016 was about three times higher than world rates [45].

Although the highest rates of CNS tumours were observed in high-middle followed by the high quintile sociodemographic index (SDI), developed countries had negative APC rates in their recent evaluations [2, 34], which comes after high APC rates in the late 20th century. Such decreasing rates might be the result of development in early diagnosis and availability of more efficient treatments for nonmalignant tumours or the separation of nonmalignant tumours in recent studies [35].

In a systematic review of primary CNS tumours in Iran from 2000 to 2009, performed by Jazayeri et al., the ASRs were 4.16 for men and 3.46 for women [23]; these are lower than the ASRs in our study which show an ascending trend. Jordan represented a total crude incidence rate of 5.01 for all malignant and nonmalignant CNS tumours in 2011 and 2012 [19].

The mean age at diagnosis was 45.54 years in our study which is higher than that of other studies in Iran [21, 23–27] and Jordan [19] but is lower than that of other developed countries which seems to be related to population structure changes. The preponderance of males among CNS tumour patients in our study is in accordance with the global estimates in 2012 (1.41 compared with 1.2 m/f ratio) [31] and the estimates in most of other studies in the world that represent male to female ratios between 1 and 2.7 [35]; however, some studies showed female predominance similar to Qom province [19, 22, 46]. The possible hypothesis for male preponderance is sex hormones and adverse working conditions such as exposure to chemicals and pesticides [47].

Our findings on the histology of tumours are not possibly conclusive enough due to notably high rates of unspecified tumours (mostly M 8000.3 and M 8001.3 codes), which are likely a reflection of lack of advanced diagnostic facilities particularly in rural areas that indicates high rates of astrocytic and oligodendroglia tumours. These findings are in line with previous studies conducted in Iran [22, 25, 27]. Lack of a unanimous tumour diagnosis even by skillful pathologists is another reason behind the high rates of unspecified tumours. Another difficulty was in the data analysis phase of the project; we found histology codes that could not be considered primary (adenocarcinomas). Thus, to avoid underestimation, such codes were first categorized into a secondary tumour group or primary/secondary group and then included in our total calculations. However, we reported primary tumours rates separately to make comparisons with other studies unchallenging for readers.

There was a noticeable difference in incidence rates in different provinces across the country; Yazd province had an ASR of 9.86 per 100000 individuals per year, and Qom province had an ASR of 1.85 per 100000 individuals per year. It showed more than five-fold difference between them, similar to the difference that was observed between different regions in the world [35] and Roshandel et al.'s study of cancer incidence in Iran in 2014 [48]. This result could likely be due to many factors such as differences in the availability of medical facilities, genetics, lifestyle, environmental, and many other probable factors that should be considered in future studies.

There is not enough information on the etiology of CNS tumours except genetics and ionizing radiations as risk factors and a possible carcinogen with unknown effects. Moreover, allergic reactions can decrease the risk of CNS tumours [49, 50]. Therefore, more comprehensive epidemiological studies are needed to find out the relationship between potential etiologies and epidemiologic diversities.

## Figures and Tables

**Table 1:** ICD-O-3 topography (site) codes.

Site	ICD-O-3 site codes
Meninges	C70.0, C70.1, C70.9
Cerebrum	C71.0
Frontal lobe of brain	C71.1
Temporal lobe of brain	C71.2
Parietal lobe of brain	C71.3
Occipital lobe of brain	C71.4
Ventricle	C71.5
Cerebellum	C71.6
Brainstem	C71.7
Overlapping lesion of brain	C71.8
Brain, NOS	C71.9
Spinal cord	C72.0
Cauda equina	C72.1
Cranial nerves	C72.2, C72.3, C72.4, C72.5
Overlapping lesion of brain and central nervous system	C72.8
Nervous system, NOS	C72.9

**Table 2:** ICD-O-3 histology codes.

Histology	ICD-O-3 histology codes
Diffuse astrocytic and oligodendroglial tumours	9380, 9382, 9400, 9401, 9411, 9440, 9441, 9442, 9450, 9451
Other astrocytic tumours	9421, 9424
Ependymal tumours	9383, 9391, 9392, 9393
Other gliomas	9430
Choroid plexus tumours	9390
Embryonal tumours	9470, 9471, 9473, 9474, 9490, 9500, 9501, 9502, 9521
Tumours of cranial and paraspinal nerves	9560, 9540
Meningioma	9530, 9531, 9534, 9537, 9538
Mesenchymal, nonmeningothelial tumours	8802, 8810, 8815, 8850, 8851, 8890, 8900, 9120, 9133, 9140, 9161, 9170, 9180, 9220, 9240, 9243, 9364, 9370, 9371
Melanocytic tumours	8720
Lymphomas	9590, 9591, 9596, 9670, 9675, 9680, 9684, 9699, 9702
Germ cell tumours	9060, 9064, 9065, 9071, 9080, 9081, 9085,
Others	9505, 9750
Unspecified	8000, 8001

**Table 3:** Distribution of tumours in different sites (topography) and patients' mean age.

Location	Total number (%)	Male mean age	Female mean age	*p* value
Meninges	408 (2.5%)	53.4	50	0.09
Cerebrum	521 (3.1%)	43.4	40.8	0.18
Frontal lobe	1316 (8%)	44.7	45.6	0.37
Temporal lobe	1039 (6.3%)	46.9	45.2	0.16
Parietal lobe	823 (5%)	47.9	46.7	0.35
Occipital lobe	182 (1.1%)	49.5	44.1	0.05
Ventricle	147 (0.9%)	35.5	38	0.47
Cerebellum	633 (3.8%)	29.2	33.1	0.03
Brainstem	182 (1.1%)	31	25.6	0.09
Brain, NOS	8968 (54.2%)	46.2	46.2	0.84
Spinal cord	515 (3.1%)	42.3	44.5	0.24
Cranial nerves	34 (0.2%)	40.4	32.2	0.36
Overlapping brain and central nervous system	45 (0.3%)	52.4	49.6	0.66
Missing	200 (1.2%)	43.7	40.5	0.32
Overlapping brain	382 (2.3%)	47	44.5	0.20
Cauda equina	7 (0.04%)	43.7	46	0.91
Nervous system, NOS	1145 (6.9%)	51.8	51.2	0.67

**Table 4:** Age-standardized incidence rate trend.

	Total			Male			Female		
Year	*N*	ASR^∗^	95% CI	*N*	ASR^∗^	95% CI	*N*	ASR^∗^	95% CI
2010	2380	3.43	3.33‐3.54	1381	3.96	3.84‐4.07	999	2.92	2.82‐3.01
2011	3243	4.64	4.52‐4.77	1927	5.51	5.39‐5.64	1316	3.78	3.67‐4.39
2012	3124	4.19	4.08‐4.31	1877	5.12	5.57‐6.09	1247	3.30	3.20‐3.40
2013	3523	4.69	4.57‐4.81	2074	5.51	5.39‐5.64	1449	3.90	3.79‐4.01
2014	4277	5.60	5.47‐5.72	2436	6.48	6.34‐6.62	1841	4.76	4.64‐4.88
Total	16547	5.19	4.99‐5.38	9695	5.56	5.41‐5.71	6852	3.93	3.81‐4.06
*p* value		<0.001		0.723				0.025	

^∗^Age-standardized incidence rate.

**Table 5:** Average crude incidence rates by histology.

	Male				Female				Total			
	*N*	%	ASR^∗^	95% CI	*N*	%	ASR	95% CI	*N*	%	ASR	95% CI
*Primary tumours*	**9695**	**95 .51**	**5.56**	**5.41-5.71**	**6852**	**95.25**	**3.93**	**3.81-4.06**	**16547**	**95.40**	**5.19**	**4.99-5.38**
Diffuse astrocytic and other oligodendroglial tumours	5144	50.68	2.88	2.79-2.98	3244	45.09	1.81	1.74-1.89	8388	48.36	2.40	2.30-2.50
Others	12	0.11	0.007	0-0.01	3	0.042	0.002	0.0001-0.005	15	0.087	0.004	0.002-0.008
Embryonal tumour	474	4.67	0.27	0.23-0.3	315	4.38	0.19	0.16-0.22	789	4.55	0.23	0.20-0.027
Meningioma	160	1.58	0.084	0.06-0.12	217	3.02	0.117	0.084-0.156	377	2.17	0.12	0.12-0.14
Mesenchymal	77	0.759	0.04	0.03-0.05	46	0.639	0.02	0.01-0.03	123	0.709	0.04	0.02-0.05
Melanocytic tumour	11	0.109	0.006	0-0.02	5	0.070	0.003	0.0001-0.015	16	0.092	0.003	0.001-0.006
Lymphomas	268	2.649	0.15	0.13-0.18	205	2.85	0.12	0.10-0.14	473	2.73	0.16	0.12-0.18
Germ cell	24	0.236	0.013	0.01-0.03	20	0.278	0.011	0.003-0.028	44	0.254	0.012	0.005-0.020
Unspecified malignancy	3130	30.83	1.87	1.78-1.95	2475	34.40	1.45	1.39-1.55	5605	32.32	2.03	1.88-2.18
Other astrocytic tumours	32	0.315	0.016	0.01-0.02	27	0.375	0.013	0.006-0.021	59	0.340	0.015	0.008-0.022
Other gliomas	8	0.079	0.005	0-0.01	7	0.097	0.003	0.0001-0.006	15	0.087	0.004	0.001-0.008
Ependymal tumour	335	3.30	0.17	0.14-0.20	273	3.780	0.13	0.11-0.16	608	3.51	0.17	0.14-0.20
Choroid plexus tumour	12	0.118	0.01	0.01-0.02	6	0.083	0.003	0.0001-0.015	18	0.104	0.006	0.001-0.011
Cranial and paraspinal nerve tumours	8	0.079	0.004	0.001-0.007	9	0.125	0.004	0.001-0.008	17	0.098	0.004	0-0.0001
*Secondary tumour*	**375**	**3.69**	**0.23**	**0.20-0.26**	**286**	**3.98**	**0.17**	**0.14-0.19**	**661**	**3.81**	**0.21**	**0.18-0.25**
*Primary or secondary tumours*	**81**	**0.798**	**0.049**	**0.04-0.06**	**56**	**0.779**	**0.031**	**0.02-0.04**	**137**	**0.790**	**0.042**	**0.027-0.057**

^∗^ASR: age-standardized incidence rate (per 10^5^ inhabitants per year).

**Table 6:** Geographical distribution of CNS tumours by provinces.

Region	Province	ASR			Number				Mean age
		Male	Female	Total	Male	Female	Total	M/F ratio	
North/North West	Ardebil	4.72	4.04	4.61	132	104	236	1.3	42.5
	Golestan	3.59	2.54	3.20	134	87	221	1.5	43.5
	Guilan	4.89	4.09	4.73	324	245	569	1.3	52.4
	Mazandaran	2.85	1.98	2.41	213	153	366	1.4	44.9
	North Khorasan	4.56	3.38	4.48	72	60	132	1.2	44.5
	East Azarbaijan	2.72	2.37	2.85	258	225	483	1.1	49.9
	Kordestan	4.29	3.64	3.95	156	124	280	1.3	43.8
	West Azarbaijan	4.01	3.09	3.54	276	221	497	1.2	46.6
	Zanjan	3.87	3.20	3.53	96	79	175	1.2	44.1

West	Hamedan	4.27	3.32	3.80	189	128	317	1.5	46
	Ilam	3.16	1.68	2.52	37	20	57	1.9	52.3
	Kermanshah	3.85	1.83	2.39	131	88	219	1.5	43
	Khouzestan	6.55	4.89	5.84	623	452	1075	1.4	43.1
	Lorestan	7.08	4.69	6.06	273	172	445	1.6	46.3

Center	Alborz	4.67	2.61	3.71	262	137	399	1.9	48.5
	Chaharmahal and Bakhtiari	3.34	2.25	2.89	65	47	112	1.4	41.4
	Qazvin	3.49	2.28	2.88	95	63	158	1.5	46.5
	Isfahan	6.87	5.27	6.35	808	568	1376	1.4	50.6
	Kerman	4.82	3.76	4.32	292	234	526	1.2	44.1
	Kohgiluyeh and Booyer Ahmad	4.84	2.89	3.87	78	39	117	2	34.9
	Markazi	8.46	6.15	7.29	305	217	522	1.4	51.9
	Semnan	5.28	4.12	4.69	79	58	137	1.4	50
	Tehran	5.33	3.71	4.52	1618	1098	2716	1.5	45.1
	Yazd	10.45	9.33	9.86	270	229	499	1.2	48.3
	Qom	1.51	2.09	1.85	43	52	95	0.8	45.9

East	Razavi Khorasan	6.31	4.74	5.59	838	615	1453	1.4	44.7
	Sistan and Baluchestan	2.75	2.12	2.44	126	91	217	1.4	37.2
	South Khorasan	3.42	2.20	2.81	48	31	79	1.5	41.5

South	Bushehr	3.83	1.76	2.83	88	36	124	2.4	40.6
	Fars	8.31	6.24	7.47	886	626	1512	1.4	46.7
	Hormozgan	2.77	2.42	2.58	91	78	169	1.2	38.9

Unknown		0.46	0.27	0.37	789	475	1264	1.7	34.1

## Data Availability

Data are available on demand for those who meet our criteria.

## Additional Points

*Key Points*. Average total age-standardized incidence rate (ASR) was 5.19 for primary tumours. The annual percent change was 13.69% during the study period. Geographical distribution showed five-fold difference in ASR among provinces.

## Supplementary Materials

Supplementary Materials Annex 1: depicts the number of patients in each age group by gender in the whole study on an age-gender pyramid. Annex 2: includes crude incidence rates by age groups and year.
