# Supplementary material for: Descriptive Epidemiology of Brain and Central Nervous System Tumours: Results from Iran National Cancer Registry, 2010-2014
Source: J Cancer Epidemiol. 2020 Sep 18;2020:3534641. doi: 10.1155/2020/3534641 (PMC7520005; doi:10.1155/2020/3534641)
Supplement: Supplementary Materials — Annex 1: depicts the number of patients in each age group by gender in the whole study on an age-gender pyramid. Annex 2: includes crude incidence rates by age groups and year. [file 3534641.f1.docx]

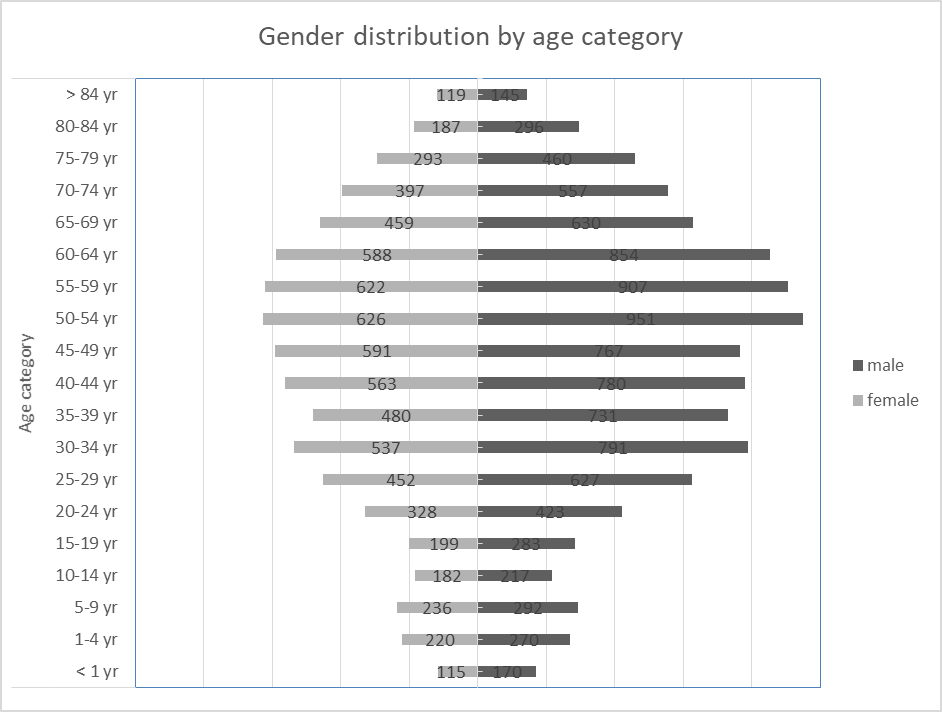


Annex 1: Gender distribution by age categories.

Annex 2: Crude incidence rates in different years of study.

|  | 2010 | | | 2011 | | | 2012 | | | 2013 | | | 2014 | | | Total | | |
| --- | --- | --- | --- | --- | --- | --- | --- | --- | --- | --- | --- | --- | --- | --- | --- | --- | --- | --- |
| Age category | N | CIR | 95% CI | N | CIR | 95% CI | N | CIR | 95% CI | N | CIR | 95% CI | N | CIR | 95% CI | N | CIR | 95% CI |
| 0-4 yr | 120 | 2.31 | 1.91-2.76 | 207 | 3.32 | 2.88-3.8 | 153 | 2.76 | 2.33-3.22 | 128 | 2.09 | 1.74-2.48 | 167 | 2.71 | 2.31-3.14 | 775 | 2.48 | 2.11-2.91 |
| 5-9 yr | 74 | 1.29 | 1.00-1.61 | 97 | 1.71 | 1.39-2.09 | 108 | 1.65 | 1.35-1.99 | 113 | 1.82 | 1.49-2.18 | 136 | 2.11 | 1.77-2.5 | 528 | 1.87 | 1.52-2.25 |
| 10-14 yr | 50 | 0.83 | 0.61-1.09 | 93 | 1.64 | 1.32-2 | 91 | 1.60 | 1.28-1.96 | 82 | 1.48 | 1.17-1.83 | 83 | 1.49 | 1.18-1.84 | 399 | 1.41 | 1.11-1.75 |
| 15-19 yr | 91 | 1.21 | 0.97-1.47 | 111 | 1.68 | 1.38-2.02 | 101 | 1.56 | 1.26-1.88 | 89 | 1.55 | 1.24-1.9 | 90 | 1.61 | 1.29-1.97 | 482 | 1.46 | 1.55-2.34 |
| 20-24 yr | 128 | 1.61 | 1.34-1.91 | 163 | 2.47 | 2.10-2.87 | 151 | 2.05 | 1.73-2.4 | 150 | 2.09 | 1.76-2.44 | 159 | 2.38 | 2.02-2.77 | 751 | 2.27 | 1.92-2.66 |
| 25-29 yr | 166 | 2.25 | 1.92-2.62 | 226 | 2.69 | 2.34-3.05 | 224 | 3.10 | 2.70-3.53 | 221 | 2.73 | 2.38-3.11 | 242 | 3.06 | 2.68-3.47 | 1079 | 2.57 | 2.23-2.93 |
| 30-34 yr | 187 | 3.21 | 2.76-3.7 | 252 | 2.91 | 2.55-3.28 | 294 | 4.78 | 4.25-5.36 | 288 | 4/00 | 3.55-4.48 | 307 | 4.13 | 3.68-4.62 | 1328 | 3.06 | 2.70-3.45 |
| 35-39 yr | 189 | 3.51 | 3.02-4.04 | 269 | 3.86 | 3.41-4.34 | 248 | 4.62 | 4.06-5.23 | 233 | 4.03 | 3.53-4.58 | 272 | 4.56 | 4.03-5.13 | 1211 | 3.47 | 3.04-3.93 |
| 40-44 yr | 182 | 3.65 | 3.14-4.22 | 284 | 5.10 | 4.52-5.72 | 259 | 4.96 | 4.37-5.6 | 300 | 5.75 | 5.11-6.43 | 318 | 5.99 | 5.34-6.68 | 1343 | 4.82 | 4.26-5.44 |
| 45-49 yr | 197 | 4.31 | 3.72-4.94 | 271 | 5.52 | 4.88-6.22 | 267 | 5.68 | 5.01-6.4 | 298 | 6.62 | 5.89-7.42 | 325 | 6.92 | 6.19-7.71 | 1358 | 5.54 | 4.90-6.24 |
| 50-54 yr | 201 | 5.26 | 4.55-6.03 | 329 | 8.16 | 7.30-9.09 | 328 | 7.66 | 6.85-8.53 | 334 | 8.20 | 7.34-9.13 | 385 | 8.94 | 8.07-9.88 | 1577 | 7.83 | 6.97-8.72 |
| 55-59 yr | 193 | 6.32 | 5.45-7.27 | 276 | 7.82 | 6.92-8.8 | 287 | 8.34 | 7.39-9.35 | 368 | 11.43 | 10.2-12.6 | 405 | 11.72 | 10.6-12.9 | 1529 | 8.67 | 7.73-9.7 |
| 60-64 yr | 177 | 8.40 | 7.19-9.72 | 263 | 9.81 | 8.66-11 | 298 | 12.04 | 10.7-13.4 | 322 | 13.02 | 11.6-14.5 | 382 | 14.08 | 12.7-15.5 | 1442 | 10.77 | 9.54-12 |
| 65-69 yr | 134 | 11.34 | 9.49-13.4 | 204 | 10.95 | 9.49-12.5 | 179 | 13.21 | 11.3-15.2 | 250 | 17.38 | 15.2-19.6 | 322 | 22.25 | 19.8-24.8 | 1089 | 11.70 | 10.2-13.3 |
| 70-74 yr | 139 | 13.35 | 11.2-15.7 | 175 | 13.02 | 11.1-15.1 | 192 | 16.45 | 14.2-18.9 | 205 | 18.14 | 15.7-20.8 | 243 | 21.28 | 18.6-24.1 | 954 | 14.20 | 12.2-16.3 |
| 75-79 yr | 89 | 11.47 | 9.20-14.1 | 122 | 10.89 | 9.04-13 | 103 | 11.35 | 9.26-13.7 | 183 | 20.84 | 17.9-24 | 256 | 29.03 | 25.5-32.8 | 753 | 13.45 | 11.4-15.8 |
| >80 yr | 80 | 10.87 | 8.61-13.5 | 92 | 10.01 | 8.06-12.2 | 108 | 12.48 | 10.2-15 | 165 | 18.92 | 16.1-22 | 302 | 33.76 | 30.0-37.7 | 747 | 16.31 | 13.81-19.14 |
| Total | 2397 | 3.27 | 3.13-3.4 | 3434 | 4.57 | 4.41-4.72 | 3391 | 4.53 | 4.38-4.68 | 3729 | 4.93 | 4.76-5.08 | 4394 | 5.74 | 5.56-5.9 | 17345 | 4.62 | 4.46-4.77 |
